# Treatment of Multiple Myeloma and the Role of Melphalan in the Era of Modern Therapies—Current Research and Clinical Approaches

**DOI:** 10.3390/jcm10091841

**Published:** 2021-04-23

**Authors:** Anastazja Poczta, Aneta Rogalska, Agnieszka Marczak

**Affiliations:** Department of Medical Biophysics, Faculty of Biology and Environmental Protection, Institute of Biophysics, University of Lodz, Pomorska 141/143, 90-236 Lodz, Poland; aneta.rogalska@biol.uni.lodz.pl (A.R.); agnieszka.marczak@biol.uni.lodz.pl (A.M.)

**Keywords:** autologous stem cell transplantation, clinical study, combination chemotherapy, high dose melphalan therapy, in vitro and in vivo studies, multiple myeloma

## Abstract

Multiple myeloma (MM) accounts for 10% of all hematological malignancies, and it is the second most common hematological neoplasm for which chemotherapy is an important pharmacological treatment. High dose melphalan followed by autologous stem cell transplantation remains the standard of treatment for transplant-eligible patients with MM. In this review, we describe aspects of the pharmacokinetics and pharmacodynamics of melphalan therapy and related compounds. In addition, we describe the use of melphalan in innovative therapies for the treatment of MM, including the development of drug carriers to reduce systemic toxicity, combination therapy to improve the effectiveness of cancer therapy, and the chemical modification of the melphalan molecule to improve antitumor activity.

## 1. Introduction

Despite constant advances in medicine, cancer remains a major health problem. It affects patients of all ages, often leading to death. Many effective anti-cancer drugs have the potential to alkylate DNA, RNA, and several proteins. DNA alkylation is the major change leading to anticancer activity. One important milestone in the fight against cancer was the discovery of nitrogen mustard as an alkylating agent in 1942. Nitrogen mustard-based DNA alkylating agents were the first effective antitumor compounds developed, and they remain important drugs for the treatment of many types of cancer. Many years of research on nitrogen mustard have resulted in the identification of a wide range of therapeutically useful compounds. Active molecules can be designed by reducing the electrophilicity of mustard agents, thereby obtaining safer analogues. This approach was used to develop clinically useful anti-cancer agents such as chlorambucil, mechlorethamine, melphalan, cyclophosphamide, and estramustine. The biological activity of this noteworthy group of compounds is based on DNA binding, cross-linking two strands, preventing DNA replication, and inducing cell cycle arrest, which leads to cell death. These alkylating agents bind to the N7 nitrogen on guanine DNA bases. DNA alkylation occurs in two stages. First, the bis(2-chloroethyl) amine undergoes first order SN2 cyclization at neutral or alkaline pH in a one-step reaction, resulting in the formation of a highly reactive and unstable aziridinium cation. In the second step, the resulting aziridine cation is subjected to nucleophilic addition by a DNA nucleophile to form a monoalkylation adduct through the SN2 mechanism. These reactions can then be repeated with another involving CH2CH2Cl to obtain a cross-link between two complementary DNA strands [1]. Many drugs and chemicals that form reactive electrophiles, including alkylating compounds, bind to cellular macromolecules such as proteins, and increase heat shock proteins synthesis by binding covalently to nucleophilic functional groups. Alkylating agents also cause secondary cytotoxic signals, such as depletion of glutathione, increased cellular calcium, oxidative stress, and lipid peroxidation, which induce a heat shock response [2].

Melphalan (MEL, trade name Alkeran™) is an alkylating drug that belongs to the nitrogen mustard group of alkylating agents. This drug was first synthesized in the second half of the 20th century. Melphalan is the phenylalanine derivative of nitrogen mustard [3]. The intracellular cytotoxic activity of melphalan is based on inter- or intra-structural DNA cross-linking and DNA-protein cross-linking via two chlororethyl groups on the molecule. These cross-links lead to deletion of nitrogen bases, strand cleavage, and open ring formation in the DNA molecule, which disrupts DNA replication and transcription. The ability of melphalan to induce both inter- and intra-strand links classifies this drug as a bifunctional alkylating agent [4].

In this review, we describe aspects of the pharmacokinetics, and pharmacodynamics of melphalan therapy and related compounds and define how melphalan is used in the treatment of multiple myeloma (MM).

## 2. Multiple Myeloma Is the Second Most Common Hematological Malignancy: Current Treatment Strategies

Plasma cell disorders are a wide group of diseases [5]. MM accounts for 1% of all malignancies, and 10% of all hematological cancers, and it is the second most common hematologic tumor after non-Hodgkin’s lymphoma [6,7]. MM is a B-cell malignancy characterized by clonal proliferation of plasma cells, overproduction of paraproteins, renal failure, hypercalcemia, anemia, osteolytic bone damage, and numerous infections [8]. Although the incidence of MM increases with age, and it is more common at 60–70 years of age, younger patients have also been diagnosed. In recent years, the median overall survival (OS) has increased from 2–3 years to 8–10 years, which is mostly due to an improved understanding of the heterogeneity of the disease, as well as the introduction of new therapeutic drugs. The use of autologous stem cell transplantation [7] or allogeneic stem cell transplantation (Allo-SCT), which is a potentially curative treatment, has also increased the survival of MM patients [9].

The treatment of MM involves different combinations of drugs with different mechanisms of action, including alkylating agents, corticosteroids, anthracyclines, immunomodulatory drugs (IMID), histone deacetylase inhibitors (iHDAC), proteasome inhibitors (PIs), monoclonal antibodies (mAbs), and high-dose chemotherapy followed by autologous stem cell transplantation (ASCT). Alkylating agents such as melphalan attack rapidly proliferating cells [7], cross-linking the two strands and arresting DNA replication, which causes cell death. In addition to melphalan, bendamustine is another alkylating agent that has been successfully used in both the upfront and relapse/refractory settings of MM patients, including those with renal impairment. This drug attracted attention because of its specific mechanism of action. Although it is structurally similar to both alkylating agents and antimetabolities, it does not show cross-resistance with alkylating drugs [10]. Glucocorticoids (especially dexamethasone), which are steroid hormones, have been used for more than 50 years in the treatment of MM. Dexamethasone works by activating the mitochondrial apoptotic pathway, upregulating pro-apoptotic genes, downregulating anti-apoptotic genes, promoting the cleavage of poly (ADP-ribose) polymerase (PARP), and activating caspase 3 [11,12,13]. The mechanistic target of rapamycin (mTOR) pathway is also involved in the mechanism of action of dexamethasone, and inhibitors of mTOR sensitize MM cells to dexamethasone-induced apoptosis [14,15]. Proteasome inhibitors such as bortezomib, carfilzomib, and ixazomib, exhibit their biological activities through various mechanisms, such as direct effects on MM cells, suppression of several adhesion molecules, inhibition of cytokines, and angiogenesis. By blocking the degradation of the kappa B inhibitor (IκB), bortezomib inhibits the NFκB signaling pathway, which plays a key role in MM cell survival and proliferation. Bortezomib inhibits the expression and secretion of vascular endothelial growth factor, thereby inhibiting angiogenesis in the bone marrow microenvironment. In addition, bortezomib inhibits oseoclasts and directly stimulates osteoblast proliferation and differentiation [7,16]. Monoclonal antibodies such as daratumumab and isatuximab bind to specific antigens on the surface of MM cells. This induces MM cell death through antibody-dependent cellular cytotoxicity (ADCC), complement dependent cytotoxicity (CDC), and/or antibody-dependent cellular phagocytosis (ADCP) [17]. Histone deacetylase inhibitors such as vorinostat and panobinostat act on malignant plasma cells by opening the chromatin structure, which leads to changes in the expression of many genes involved in signaling pathways, cell cycle inhibition, and angiogenesis. This leads to cell growth arrest, activation of external, and/or internal apoptotic pathways, induction of autophagy, activation of mitotic cell death, and senescence [18,19]. A promising agent for the treatment of patients with relapsed or refractory MM is venetoclax (ABT-199), a selective, orally bioavailable B cell lymphoma 2 (BCL-2) inhibitor. It is particularly effective in MM harboring t(11;14), which is characterized by high expression of BCL-2 relative to B-cell lymphoma-extra large (BCL-XL) and myeloid cell leukemia-1 (MCL-1) [20,21]. Immunomodulatory drugs such as thalidomide, lenalidomide, and pomalidomide modulate the inflammatory environment of the bone marrow, causing MM cell death by inhibiting angiogenesis and antiproliferative properties [22]. Cereblon (CRBN) is a target for immunomodulatory drugs [23], and lenalidomide-bound cereblon acquires the ability to target two specific B cell transcription factors, Ikaros family zinc finger proteins 1 and 3 (IKZF1 and IKZF3), for proteasomal degradation [24]. Another promising treatment for MM is anti-B cell maturation antigen (BCMA) chimeric antigen receptor (CAR) T cell therapy. It has shown improved efficacy with the bivalent BM38 CAR-T therapy for relapsed/refractory MM with a high overall response rate (ORR) [25].

## 3. Pharmacokinetics and Pharmacodynamics of Melphalan

The pharmacokinetic parameters of melphalan were tested in several research centers [26,27,28,29,30,31,32], and the results showed large interindividual differences in the parameters analyzed. The dominant half-life (t1/2β) values range between 25 and 96 min, and the melphalan plasma clearance is 127–797 mL/min/m^2^ [26,27]. The average volume of distribution ranges from 6 to 108 L/m^2^ [28,29,30]. Research of melphalan administered at a high dose (180 mg/m^2^) shows that plasma levels of melphalan decline bi-exponentially, with a mean terminal half-life (t1/2β) of 61 min (range 40.3–132.8 min). The estimated peak concentrations are 5.45–16.57 mcg/mL. The average volume of distribution at steady state and clearance are 0.479 ± 0.164 L/kg and 6.73 ± 1.60 mL/min/kg, respectively. These kinetic parameters are similar to those observed for lower doses of melphalan [33]. Melphalan administered in oral form is rapidly absorbed after administration. Absorption lag-time is <1 h [34]. Alberts et al. showed that oral melphalan has a mean plasma terminal phase half-life (t1/2) of 90 ± 17 min. The mean area under the plasma concentration: time curve (CXT) is 53 ± 33 µg/ min/mL. Urinary excretion averages 10.9 ± 4.9% during the first 24 h. The average CXT ratio (oral: intravenous) is 0.56 (range, 0.25–0.89) [35]. A large variation in bioavailability between individuals has been observed after p.o. treatment. Although this parameter is not dependent on the dose administered, it decreases with the duration of the treatment. This suggests that it may be advantageous to administer oral melphalan for fewer days to achieve higher bioavailability. Absorption of melphalan is consistent with first order kinetics at the dose intervals tested [34]. Reece et al. [36] confirmed that to achieve the best bioavailability, melphalan should be administered on an empty stomach, as administration with food, especially fat food, reduces the melphalan exposure (AUC) by up to 39%.

Melphalan enters the cells mainly via the neutral leucine active amino acid pathway [37]. Studies using murine L12106 leukemia cells show that the transport of melphalan is mediated equally by two separate amino acid transport systems: one system is mediated by a monovalent-dependent cation that has the highest affinity for leucine, and the second is the L system, the classic leucine-preferable sodium independent transport system. The model synthetic substrate for the L system is 2-aminobicyclo[2.2.1]heptane-2-carboxylic acid (BCH) [1]. Identical carrier systems have been identified for L-PAM transport in LPC-1 plasmacytoma cells and L5178Y lymphoblasts [38,39]. Studies on the mechanism of melphalan uptake by L5178Y lymphoblasts have been extended by focusing on the chemical specificity of the transport mechanism.

Melphalan uptake is an active carrier mediated process. It proceeds “uphill” against a concentration gradient, is temperature-sensitive, partly sodium-dependent, and is inhibited by several metabolic antagonists. Studies indicate that melphalan uptake follows Michaelis-Menten two-phase kinetics, suggesting the involvement of at least two carrier systems, and it is inhibited by various amino acids [40,41]. A strong inhibitor of melphalan uptake is β-2-aminobicyclo[2.2.1]heptane-2-carboxylic acid, a specific inhibitor of the L-amino acid transport system (preferring leucine), but not by 2-aminoisobutyric acid or 2-(methylamino)-isobutyric acid-specific inhibitors of the amino acid system A (preferring alanine). Under conditions of full saturation of the L and A systems with β-2-aminobicyclo[2.2.1]heptane-2-carboxylic acid and 2-aminoisobutyric acid, drug uptake is inhibited by serine, an amino acid that is transported through the ASC system (alanine, serine and cysteine). In experiments using leucine as a substrate, melphalan inhibited the uptake of the amino acid by both the L system and the second ASC-like system. Carrier-dependent melphalan uptake can be explained by transport through these two systems. When the drug concentration range is 3.33–20 µM, the carrier-mediated uptake of melphalan is mediated equally by the L system and the ASC system, whereas in the 20–100 µM concentration range, the L system becomes increasingly dominant [40].

Large neutral amino acid transporter 1 (LAT1 or SLC7A5) is a sodium- and pH-independent transporter that provides vital amino acids (e.g., leucine and phenylalanine) to cells. Melphalan is transported to the brain via cerebrovascular LAT1, demonstrating the usefulness of LAT1 for drug delivery in the central nervous system. This drug has strong structural similarity to endogenous LAT1 substrates. Prodrugs directed at LAT1 show structural similarity: they are composed of a parent drug attached to the amino acid side chain via a biodegradable bond and an unsubstituted α-carboxyl and α-amino group to achieve effective LAT binding [42].

Melphalan is eliminated both by the kidneys and by spontaneous chemical degradation to mono- and dihydroxy metabolites. The latter pathway has a relatively small share (just over 5%) because plasma protein binding delays the rate of melphalan hydrolysis. However, melphalan is degraded rapidly in the urine, which leads to a very variable dose percentage that can be recovered from urine within 24 h. This led to some confusion about the role of kidney function in the elimination of melphalan. The fact that more than 60% of the dose was recovered in three of nine patients in one study suggests that renal excretion is the main route of melphalan elimination [43].

## 4. Autologous Stem Cell Transplantation (ASCT) in Combination with High Doses of Melphalan (HDM) as the Standard Treatment for Newly Diagnosed Patients with Multiple Myeloma

The current treatment regimen for newly diagnosed MM (NDMM) involves obtaining the deepest remission of the disease followed by maintenance of this response through continuous therapy [44,45].

Initial use of high-dose melphalan (HDM) results in a higher response; however, the high toxicity associated with bone marrow recovery time outweighs the benefits. The combination of autologous stem cell infusion with HDM reduces toxicity and leads to better outcomes. HDM-ASCT for the treatment of MM was first developed by Barlogie, McElwain, and others in the mid-1980s and was the first treatment milestone leading to better outcomes [3,46]. In the age of modern therapy, HDM-ASCT remains the standard approach for the treatment of patients with newly diagnosed MM who are eligible for transplantation [44,47,48]. High-dose therapy combined with ASCT is considered to be the standard of care for MM patients <65 years of age. MM is a disease of the elderly, as the median age at diagnosis is 70 years. Therefore, a large proportion of patients are considered ineligible for high-dose therapy because of increased treatment-related toxicity and mortality associated with a melphalan dose of 200 mg/m^2^ [49]. However, high-dose therapy with melphalan 200 mg/m^2^ is safe in selected elderly patients with NDMM, even in those older than 70 years without increased mortality [50].

Randomized trials comparing HDM-ASCT with conventional chemotherapy have demonstrated the clinical benefit of HDM-ASCT. HDM-ASCT was compared with various chemotherapy combinations including doxorubicin, vincristine, melphalan, cyclophosphamide, carmustine, and prednisone [47,48]. An overview of phase III clinical trials comparing the MM regimen with or without ASCT is provided in Table 1.

In 1996, Attal et al. [51] published the first such studies on NDMM. The study showed that high-dose melphalan therapy combined with ASCT improves response rate, event free survival, and OS in patients with myeloma [51]. Another randomized, multicenter phase III study conducted by Attal et al. [52] in patients with MM showed that treatment with lenalidomide, bortezomib, and dexamethasone (RVD) plus ASCT resulted in longer progression-free survival (PFS) than RVD alone; however, OS did not differ significantly between the two treatment arms [52].

The use of ASCT as an intensification therapy and consolidation therapy in patients with NDMM compared with new therapies (bortezomib-melphalan-prednisone, with or without bortezomib-lenalidomide-dexamethasone consolidation therapy and lenalidomide-dexamethasone maintenance) was supported by a multicenter, randomized, open-label phase III study conducted by the team of Prof. Cavo [53]. The results of the study support the use of ASCT as intensification therapy and the use of consolidation therapy in patients with NDMM, even in the era of novel treatments. PFS, but not OS, was significantly improved with ASCT compared with VMP (bortezomib–melphalan–prednisone) [53].

In a randomized, phase III study by Gay et al. [54] in patients with NDMM, consolidation of chemotherapy (cyclophosphamide and dexamethasone) with lenalidomide significantly increased the risk of progression or death and decreased OS compared with HDM-ASCT. The results of this study confirmed that consolidation with HDM-ASCT remains the preferred therapeutic option in transplant-eligible patients with NDMM. This regimen improves PFS and OS at the expense of increased, but manageable, adverse events. A phase III randomized trial by Palumbo et al. [55] compared melphalan 200 mg and ASCT with melphalan, prednisone and lenalidomide (MPR); the results favored melphalan and ASCT. Both PFS and OS were significantly longer with HDM-ASCT than with MPR [55].

More than 30 years after the introduction of ASCT to the therapy of patients with multiple myeloma, there are steel studying at different aspects of ASCT as: early or delayed, single or tandem [56,57]. The randomized, open-label phase III study BSBMT/UKMF Myeloma X Relapse showed that salvage ASCT increases OS during consolidation of reinduction treatment in patients with MM at first relapse following the first ASCT. Delaying salvage ASCT to third-line treatment or later may not be as beneficial as using salvage ASCT at first relapse [58].

For over 10 years, numerous studies have compared single and tandem ASCT with melphalan conditioning [59]. Tandem ASCT refers to the re-administration of ASCT within 6 months of the first application. Patients randomly assigned to a second autologous hematopoietic cell transplantation (AHCT/AHCT + lenalidomide) received high-dose melphalan (200 mg/m^2^) followed by autologous peripheral-blood stem-cell infusion [59]. In another study of tandem transplantation, the second high-dose regimen was administered at 140 mg/m^2^ [60]. Despite numerous clinical trials, tandem ASCT remains controversial and is recommended for patients who did not achieve a very good partial response (VGPR) after the first ASCT or NDMM patients with high-risk disease characteristics, including patients with high-risk cytogenetics [57,59].

The Phase III BMT CTN 0702 study was designed to improve PFS by comparing ASCT, tandem ASCT, and ASCT with four consecutive cycles of RVD. The results showed that a second consolidation of ASCT or RVD as post-ASCT interventions in the initial treatment of transplant-eligible MM patients did not improve PFS or OS. A single ASCT and lenalidomide should remain the standard approach [59]. The latest clinical trials involving new MM treatment regimens include the use of HDM-ASCT (Table 2).

More than 30 years after its introduction, HDM-ASCT remains in the arsenal of therapy for patients with newly diagnosed MM. Many clinical trials are currently underway to assess the efficacy of combination therapy with melphalan for the treatment of MM (Table 3). Novel therapies represent a milestone in the treatment of MM, and have contributed to a significant increase in the survival of MM patients over the past two decades [48,61,62].

**Table 1:** Clinical trials assessing the efficacy of combination therapy for the treatment of MM, including melphalan-new directions. Overview of phase III clinical studies comparing the MM treatment regimen with or without Autologous Stem Cell Transplantation.

Ref.	Type of Study	No. of Patients	Treatment Regimen	Results			
				Response	PFS	OS	MRD Negativity
[53,61]	Multicenter, randomized, open-label, phase III study	1503	I: MEL (200 mg/m^2^) + ASCT (intensification therapy) + RVD/no cons.	VGPR: 84%	56.7 months (95% CI 49.3–64.5)	NA	36% (10-5)
			II: VMP (intensification therapy) + RVD/no cons.	VGPR: 75%	41·9 months (95% CI 37.5–46.9)	NA	64% (10-5)
				HR for PFS of ASCT compared with VMP: 0.73, 0.62–0.85; *p* = 0.0001.			
[55,61]	Open-label, randomized, phase III study	402	I: MEL (200 mg/m^2^) + ASCT (consolidation therapy) ± Rm.	CR (post-consolidation): 23%	43.0 months	4-year 81.6%	NA
			II: MPR (consolidation therapy) ± Rm.	CR (post-consolidation): 18%	22.4 months	4-year 65.3%	NA
				HR for PFS: 0.44; 95% CI: 0.32–0.61; *p* < 0.001. HR for OS: 0.55; 95% CI, 0.32–0.93; *p* = 0.02.			
[54,61]	Multicenter, randomized, open-label, phase III study	389	I: MEL (200 mg/m^2^) +ASCT (consolidation therapy) +Rm./RPm.	CR: 33% (MEL-ASCT +Rm.) CR: 37% (MEL-ASCT +RPm.)	43.3 months (95% CI 33.2–52.2);	4-year OS: 75% (MEL-ASCT + Rm.) 4-year OS: 77% (MEL-ASCT + RPm.)	NA
			II: CRD (consolidation therapy) +Rm./RPm.	CR: 27% (CRD + Rm.)CR: 23% (CRD + RPm.)	28.6 months (95% CI 20.6–36.7)	4-year OS: 77% (CRD + Rm.) 4-year OS:76% (CRD + RPm.)	NA
				HR for the first 24 months 2.51, 95% CI 1.60–3.94; *p* < 0.0001			
[59]	Prospective, randomized, phase III study	758	I: MEL+ ASCT (consolidation therapy) + Rm.	1-year ORR: 47.1% (*n* = 208)	53.9% (95% CI: 47.4–60%)	38-month OS: 83.7% (95% CI: 78.4–87.8%)	NA
			II: MEL+ ASCT/ASCT (consolidation therapy) + Rm.	1-year ORR: 50.5% (*n* = 192)	58.5% (95% CI: 51.7–64.6%)	38-month OS: 81.8% (95% CI: 76.2–86.2%)	NA
			III: MEL+ ASCT +RVD (consolidation therapy) +Rm.	1-year ORR: 58.4% (*n* = 209)	57.8% (95% CI: 51.4–63.7%)	38-month OS: 85.4% (95% CI: 80.4–89.3%)	NA
				Patients with high-risk disease experienced higher rates of treatment failure (progression or death; HR, 1.66; 95% CI: 1.30–2.11) and overall mortality (HR, 1.49; 95% CI: 1.01– 2.20) compared with patients with standard-risk disease.			
[52,61]	Open-label, randomized, phase III study	700	I: MEL (200 mg/m^2^) + ASCT+ RVD (consolidation therapy) + Rm.	CR: 59%	50 months	4-year OS: 81%	79% (10-4)
			II: RVD (consolidation therapy) +Rm.	CR: 48%	36 months	4-year OS: 82%	65% (10-4)
				HR for disease progression or death, 0.65; *p* < 0.001			
[58,63]	Open-label, randomized, phase III study	297	I: MEL (200 mg/m^2^) + sASCT (consolidation therapy)	CR: 92.1%	19 months (95% CI 16–26)	67 months (95% CI 55–not estimable)	NA
			II: cyclophosphamine (consolidation therapy)	CR: 94.1%	11 months (95% CI: 9–12)	52 months (95% CI 42–60)	NA
				HR for PFS: 0.45 (95% CI 0.31–0.64), *p* < 0.0001 HR for OS: 0.56 (0.35–0.90), *p* = 0.0169			

Abbreviations: ASCT, autologous stem cell transplantation; sASCT, salvage autologous stem cell transplantation; CR, complete remission; CRD, cyclophosphamide + lenalidomide + dexamethasone; HR, hazard ratio; MM, multiple myeloma; MPR, melphalan + prednisone + lenalidomide; MRD, minimal residual disease; NA, not available; ORR, overall response rate; OS, overall survival; PFS, progression-free survival; Rm, lenalidomide maintenance; RPm, lenalidomide + prednisone maintenance; RVD, lenalidomide + bortezomib + dexamethasone; VGPR, very good partial response; VMP, bortezomib + melphalan + prednisone.

**Table 2:** Clinical trials assessing the efficacy of combination therapy for the treatment of MM, including melphalan-new directions. Published clinical studies featuring new MM treatment regimens with Autologous Stem Cell Transplantation.

Ref.	Type of Study	No. of Patients	Treatment Regimen	Results
[64]	Randomized, a double-blind, placebo-controlled phase III trial	656	I: ixazomib maintenance therapy II: placebo both groups had undergone standard induction therapy with MEL (200 mg/m^2^) conditioning and a single ASCT	There was a 28% reduction in the risk of PFS with ixazomib vs. placebo (26.5 months (95% CI 23.7–33.8) vs. 21.3 months (18.0–24.7); HR 0.72, 95% CI 0.58–0.89; *p* = 0.0023). At the time of this analysis no increase in secondary malignancies was observed with ixazomib therapy (3% patients) compared with placebo (3% patients).
[65,66,67]	Open-label, randomized, phase III study	458	RVD (induction therapy) + BU (12 mg/kg)- MEL (140 mg/m^2^) + ASCT /MEL (200 mg/m^2^) +ASCT + RVD (consolidation therapy)	Conditioning with BU-MEL in comparison to MEL was associated with longer PFS (41 vs. 31 months; *p* = 0.009), although OS was similar to that in the melphalan 200 mg/m^2^ group. This should be counterbalanced against the higher frequency of veno-occlusive disease-related deaths. Access to novel agents as a salvage therapy after relapse/progression was decreased for patients receiving BU-MEL (43%) vs. MEL (58%; *p* = 0.01).
[68]	Prospective, investigator-initiated, nonrandomized, multicenter, open-label, phase II study	100	RVD (induction therapy) + MEL (200 mg/m^2^) + ASCT + Rm ± PCD	PCD was an effective therapy after first relapse with RVD. Responses were obtained in 85% of patients evaluated: CR (1%), VGPR (33%).After 4 cycles, the rate of PR (or better) was 85%. 94% of planned ASCTs were performed.
[69]	Single-arm, prospective phase II study	125	I: MEL (200 mg/m^2^) + ASCT + Lipegfilgrastim (LIP) II: MEL (200 mg/m^2^) + ASCT + Filgrastim (FIL)	The median duration of grade 4 neutropenia was 5 days in both LIP and FIL groups. The incidence of FN was significantly lower in the LIP than in the FIL group (29% vs. 49%, respectively, *p* = 0.024). The HR of ANC ≥ 0.5 × 10(9)/L was 3.5 times higher in patients treated with LIP than in those treated with FIL (HR 3.50, 95% CI 2.28–5.38, *p* < 0.001), indicating that the response was faster in LIP treated patients than in those treated with FIL.

Abbreviations: ANC, absolute neutrophil count; ASCT, autologous stem cell transplantation; BU-MEL, busulfan + melphalan; CR, complete remission; FN, febrile neutropenia; HR, hazard ratio; MM, multiple myeloma; OS, overall survival; PCD, pomalidomide + cyclophosphamide + dexamethasone; PFS, progression-free survival; PR, partial remission; Rm, lenalidomide maintenance; RVD, lenalidomide + bortezomib + dexamethasone; VGPR, very good partial response.

**Table 3:** Clinical trials assessing the efficacy of combination therapy for the treatment of MM, including melphalan-new directions. New clinical studies of the treatment of MM with combination therapy including melphalan.

Clinical Trial Identifier	Trial Phase	Treatment Regimen	Objective of Trial
NCT03829371	1	VMP, MPT and lenalidomide with low-dose dexamethasone	Comparison of treatment regimens in an autologous stem cell transplantation ineligible population affected by MM.
NCT03346135	2	melphalan, daratumumab	Daratumumab after stem cell transplant for the treatment of MM.
NCT03481556	2	melphalan, dexamethasone, bortezomib, daratumumab	Assessing patients with relapsed or relapsed-refractory MM following 1–4 lines of prior therapy.
NCT04466475	1	astatine at 211 anti-cd38 monoclonal antibody okt10-b10, melphalan	Radioimmunotherapy and chemotherapy before stem cell transplantation. Therapy based on 211At-OKT10-B10 in combination with melphalan before a stem cell transplant may be more effective than melphalan monotherapy in MM.
NCT03556332	1	carfilzomib, lenalidomide, dexamethasone, daratumumab, Procedure: autologous hematopoietic cell transplantation (melphalan)	Assessing patients with relapsed or refractory myeloma with re-administration of ASCT to a patient with symptoms of disease progression. The effect of the drugs in combinations will be compared before and after ASCT in MM.
NCT02581007	2	fludarabine, melphalan, cyclophosphamide	Evaluation of the safety and efficacy of a reduced intensity allogeneic HSCT from partially HLA-mismatched first-degree relatives utilizing PBSC as the stem cell source.
NCT04008888	1	melphalan, fludarabine, PI and dexamethasone as maintenance therapy, PI + IMids + dexamethasone as consolidated chemotherapy	Assessing efficacy and safety of the holistic treatment of young high-risk MM patients who were designed to receive a combination of high-dose chemotherapy with allogeneic or autologous HSCT.
NCT01453088	3	melphalan, bortezomib	Assessing a standard regimen and the newly established melphalan and bortezomib regimen in patients with MM 65 years or older.
NCT02780609	½	selinexor, melphalan, dexamethasone, fosaprepitant	Determination of the maximum tolerated dose of selinexor in combination with high-dose melphalan as a conditioning regimen for hematopoietic cell transplant in MM.
NCT03570983	2	allopurinol, carmustine, etoposide, cytarabine, melphalan	Comparing melphalan to carmustine, etoposide, cytarabine, and melphalan (beam) as a conditioning regimen for patients with MM undergoing high dose therapy followed by autologous stem cell reinfusion.
NCT02043847	1	radiation: total marrow irradiation drug:melphalan, filgrastim (g-csf)	Assessing patients with relapsed or refractory MM will receive high dose melphalan with autologous stem cell rescue. The pre-transplant conditioning is based on total marrow irradiation.

Abbreviations: ASCT, autologous stem cell transplantation; HSCT, hematopoietic stem cell transplantation; IMID, immunomodulatory drugs; MM, multiple myeloma; MPT, melphalan-prednisone- thalidomide; PBSC, peripheral blood stem cell; PI, proteasome inhibitors; VMP, bortezomib + melphalan + prednisone.

## 5. Clinical Usage of Combination Treatment with Melphalan to Improve the Effectiveness of Cancer Therapy

Oral administration of melphalan and prednisone as an immunosuppressant against MM was first described by Alexanian et al. in 1969 [70]. This combination resulted in an increase in the response rate and median survival of 6 months compared with melphalan alone [37].

The introduction of novel therapies is an important milestone in the treatment of MM that has markedly increased the survival of MM patients over the last two decades. The immunomodulatory drug thalidomide and its lenalidomide derivative, and the proteasome inhibitor bortezomib have improved the natural history of MM. The usage and optimization of the combination of these drugs have improved the OS of patients with MM. These drugs are currently included as induction and maintenance therapy [37,48].

The aim of induction treatment of MM patients eligible for transplantation is to obtain the earliest possible response for rapid disease control and the maximum possible response without excessive toxicity to safely enter ASCT. Three-drug combinations including a PI with an IMiD and dexamethasone are currently considered as the gold standard regimens [48,71]. The combination of bortezomib, thalidomide, and dexamethasone (VTD) shows superiority over the combinations of the two drugs thalidomide-dexamethasone (TD) and bortezomib-dexamethasone (VD) in terms of response rates and long-term outcomes [56,72,73]. Lenalidomide in combination with bortezomib and dexamethasone (RVD) has advantages over lenalidomide-dexamethasone (RD) and is associated with deeper and sustained responses and increased survival [74]. RVD is also associated with improved OS compared with the combination of bortezomib, cyclophosphamide and dexamethasone (VCD) [75]. Induction therapy with RVD showed high rates of deep response in the Phase III clinical trial, as more than one-third of NDMM patients eligible for transplantation were minimal residual disease (MRD) negative. RVD therapy has become the predominant induction regimen in the United States, although VTD or even VCD are feasible options depending on drug availability [74,76].

Another approach for patients with newly diagnosed MM who are not eligible for ASCT is the inclusion of daratumumab in standard therapy. Daratumumab is a human IgGκ monoclonal antibody against a CD38 cell surface marker that is expressed on the surface of hematopoietic cells, and is overexpressed on MM cells. CD38 acts as a receptor and as an ectoenzyme, thereby performing many functions, and its multi-faceted mechanisms of action include direct antitumor and immunomodulatory activity [17,77,78]. Daratumumab may also sensitize myeloma cells to other drugs by decreasing CD38 expression levels and/or restoring depleted T cell responses [78,79]. Combination therapy consisting of intravenous administration of daratumumab, bortezomib, melphalan, and prednisone (Dara-VMP) in patients with newly diagnosed MM who are not eligible for ASCT has been approved based on the results of the phase III ALCYONE trial. This therapy significantly extended the median PFS compared with therapy without daratumumab [77,80,81]. The MAIA study (NCT02252172) confirmed the efficacy and safety of daratumumab in NDMM patients who were not eligible for ASCT, although it compared the use of daratumumab in combination with lenalidomide and dexamethasone (Dara-RD) vs. lenalidomide and dexamethasone alone. The results of this phase III trial showed that treatment with daratumumab plus lenalidomide and dexamethasone results in significantly longer PFS than lenalidomide and dexamethasone alone; the risk of disease progression or death was 44% lower in the daratumumab group than in the control group. The addition of daratumumab improved the efficacy of both VMP and RD [82]. Choosing between Dara-VMP and Dara-RD can be difficult because there is currently no direct comparison of the two combinations. In Italy, a study performing a head-to-head comparison of VMP vs. RD (NCT03829371) is underway [83]. New melphalan treatment regimens are constantly being developed and are currently in the early stages of clinical trials (Table 3). The proposed therapies are, among others, based on the next-generation proteasome inhibitor carfilzomib (Kyprolis^®^) (NCT03556332) and the exportin 1 inhibitor selinexor (NCT0278 0609). Exportin 1 is overexpressed 2- to 4-fold in MM. Despite considerable advances, there are still problems with systemic toxicity, which hampers optimal VMP administration and extends the duration of treatment. Carfilzomib is a proteasome inhibitor that selectively and irreversibly binds to the constitutive proteasome and immunoproteasome. In a preclinical model, carfilzomib showed a stronger anti-myeloma effect than bortezomib. In addition, this new generation proteasome inhibitor has a different safety profile than bortezomib, showing a very low incidence of neuropathy [84]. The side effects of drugs were described in the phase III ENDEAVOR clinical trial. This study compared the safety profiles of the two regimens, carfilzomib and dexamethasone and bortezomib and dexamethasone. The safety profiles were similar, although the carfilzomib group showed a higher number of grade 3 adverse events and serious adverse events; however, these were deemed to be manageable and may be accounted for by the longer average treatment period than that of the bortezomib group [85].

## 6. “Weak Side” of Melphalan

High-dose therapy is burdened by plenty of side effects, significant morbidity, and rarely, treatment-related mortality [86]. Melphalan-induced side effects depend strongly on the dose [87]. HDM-ASCT leads to high-grade toxicities such as prolonged bone marrow suppression, nausea, vomiting [88], diarrhea, alopecia, rash, pruritus, mouth ulceration, hypersensitivity reactions [89], mucositis [67,90], infections (bacteremia, pneumonia, *Clostridium difficile*, fungal infection, sepsis, septic shock), vascular disorders, and thromboembolic events (pulmonary embolism, ischemic cardiopathy, ischemic stroke) [52]. Uncommon but potentially serious side effects include veno-occlusive disease, autologous graft-versus-host disease, graft failure [86], irreversible myelosuppression, hemolytic anemia, pulmonary fibrosis, anaphylaxis [89], nutrition problems, and weight loss [91].

A common side effect of high-dose melphalan therapy is cardiotoxicity, which is manifested as supraventricular tachycardia and atrial fibrillation [92,93]. The use of a high concentration of melphalan in myeloablative therapy in preparation for hematopoietic cell transplantation is highly hepatotoxic, as it is associated with high enzyme growth rates and acute liver damage due to sinusoidal obstruction syndrome. In most patients, serum aminotransferase levels increase markedly (5–20 times the normal upper limit) [89].

**Figure 1:**
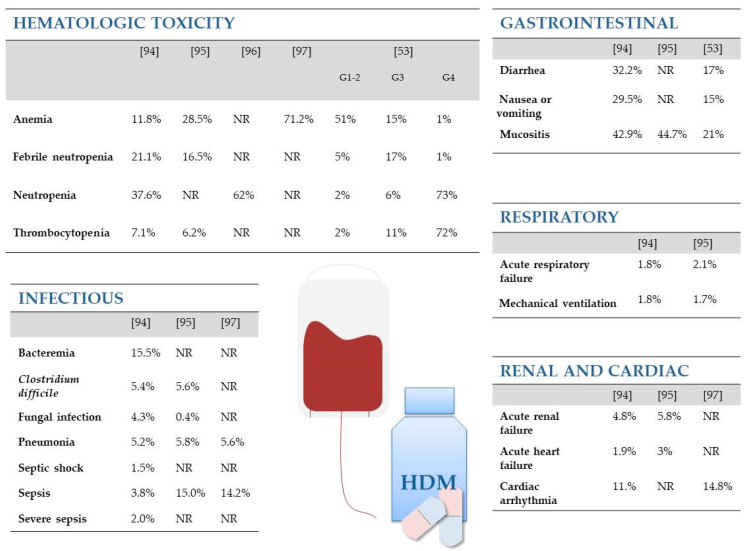
Side effects of HDM-ASCT therapy include: hematologic toxicity, infection, gastrointestinal complaints, pulmonary disease, acute renal failure, and cardiac arrhythmias. Figure based on [53,94,95,96,97]. G-grade (adverse events grades 1–2 occurring in at least 10% of patients and adverse events grades 3–4 in all patients [53]); NR, not reported.

A population-based study that aimed to determine in-hospital mortality and complications after ASCT showed that elderly patients (>65 years) are at increased risk of complications after transplantation, including severe sepsis, acute respiratory failure, septic shock, pulmonary disease, acute renal failure, cardiac arrhythmias, and prolonged mechanical ventilation compared with patients under 65 years of age. In-hospital mortality in MM patients following ASCT is rare (1.5%), and in in-hospital mortality does not differ significantly between elderly and younger patients [94,95] (Figure 1).

## 7. Drug Resistance to Melphalan

Multiple drug resistance (MDR) contributes to the failure of cancer treatment leading to clinical relapse. MDR is the phenomenon by which cancer cells become resistant to a wide variety of unrelated drugs after exposure to a single chemotherapeutic agent. Despite advancements in MM treatment, drug resistance develops frequently during the antimyeloma therapy [98]. Melphalan is administered at low concentrations for initial therapy of patients that are not eligible for ASCT and is, at high concentration, the most common conditioning treatment for patients undergoing ASCT. VMP data from 59 patients newly diagnosed with MM were collected and analyzed. Of these patients, 78% received 9-cycle regimens. As many as 84% of patients underwent a reduction in the dose of drugs during the cycles. There were no statistically significant differences in PFS and OS between the high dose (≥52.1 mg/m^2^) and low dose (<52.1 mg/m^2^) groups. The reason for reducing the dose of drugs in patients was non-hematological toxicity (92.7%) including peripheral neuropathy (36.6%). Chromosomal abnormalities were identified in 17 (28.8%) patients [99]. On the other hand, new clinical studies suggest that combination therapies may overcome drug resistance and may have additive or even synergistic effects with melphalan. In the phase III ALCYONE study, melphalan, as one of the drugs in the Dara-VMP regimen, was administered orally at a dose of 9 mg/m^2^, once daily on days 1–4 of each cycle. Treatment with this combination led to grade 3 or 4 infection-related side effects and adverse infusion reactions despite increasing the OS of MM patients [100].

High doses of melphalan, as well as low doses administered over a long period of time, can lead to the development of drug resistance. A commonly accepted practice before ASCT is the administration of high doses of melphalan. A study analyzed 27 patients with advanced MM who received 220 mg/m^2^ i.v. melphalan (HDM220) followed by ASCT. The study group consisted of nine patients with primary refractory disease and 18 patients who relapsed after responding to the previous high-dose therapy. In the group of patients who had previously received intensive care and then relapsed, high-dose melphalan was effective only when the disease was chemosensitive. In patients with relapsed disease that showed resistance to treatment, increasing the melphalan dose was ineffective, with an event-free survival (EFS) rate of 0% after 1 year. The major adverse side effect was grade 4 mucositis in 63% of patients [101]. Another study analyzed 1964 patients to determine whether melphalan 200 mg/m^2^ and melphalan 140 mg/m^2^ are equally effective and tolerable at first single autologous transplantation episodes. Studies show that the disease state at the time of transplantation affects OS and PFS. These indicators were significantly greater in patients with poor clinical responses to induction therapies who received melphalan at a dose of 200 mg/m^2^. Research has also shown that transplantation in patients with very good partial or complete response significantly preferred melphalan 140 mg/m^2^ for OS (adjusted hazard ratio: 2.02) [102]. However, resistance to melphalan can occur and can lead to relapse after ASCT, and early relapse results in reduced survival [103]. Another study identified an association between polymorphisms of genes involved in DNA repair and melphalan resistance in MM. In a group of MM patients treated with high-dose melphalan and ASCT, single nucleotide polymorphisms of Poly (ADP-ribose) Polymerase *(PARP)*, RAD51 Recombinase *(RAD51)*, Proliferating Cell Nuclear Antigen *(PCNA)*, 8-Oxoguanine DNA Glycosylase *(OGG1),* Xeroderma Pigmentosum, Complementation Group C (*XPC*), Breast And Ovarian Cancer Susceptibility Protein 1 *(BRCA1)*, Excision Repair 1, Endonuclease Non-Catalytic Subunit (*ERCC1*), *BRCA1* Associated RING Domain 1 *(BARD1*), and Tumor Protein P53 Binding Protein 1 *(TP53BP1*) were associated with the outcome and OS of patients [104]. *ERCC2* and *XRCC3* gene polymorphisms are also associated with treatment outcome and drug resistance in patients treated with high-dose melphalan and ASCT [105]. Moreover, a combination of IMiD followed by HDM-ASCT leads to adverse outcomes associated with somatic mutations in the peripheral blood named clonal hematopoiesis of indeterminate potential (CHIP). In a study of 629 MM patients treated by ASCT, CHIP was detected in 136/629 patients (21.6%). Cell sequencing indicated a mutation mainly of DNA Methyltransferase 3 Alpha *(DNMT3A),* Tet Methylcytosine Dioxygenase 2 *(TET2),* Tumor Protein P53 *(TP53),* Additional Sex Combs Like 1, Transcriptional Regulator *(ASXL1)*, and Protein Phosphatase, Mg2+/Mn2+ Dependent 1D *(PPM1D)* genes, which were associated with a significantly reduced PFS and OS as compared to patients without CHIP. It is suggested that the presence of CHIP might be associated with worse outcomes, which indicates the benefit of performing research in this direction to newly diagnosed MM patients before ASCT [106].

A few mechanisms of resistance to melphalan have been described. A study reported that MM cells from patients previously treated with melphalan can repair DNA crosslinks in vitro [107]. DNA repair in the course of leukemia occurs mainly through the base excision repair and Fanconi anemia (FA)/BRCA repair pathways [108]. DNA damage in peripheral blood mononuclear cells is a predictor of clinical outcome in patients treated with high-dose melphalan and ASCT [109]. Moreover, genetic lesions affecting both alleles of the tumor suppressor gene *TP53* are major indicators of unfavorable prognosis in newly diagnosed MM [110]. Only 3.7% of patients are diagnosed with biallelic changes in the *TP53* gene in the form of a loss or mutation (called double-hit myeloma) [111]. By contrast, in a cohort of patients with relapsed MM, *TP53* abnormalities were identified in 45% of the patients, and the double hit event del(17p)/TP53mut or del(17p)/TP53del was present in 15% of the cases [112,113]. Second hits (del17p+ TP53 point mutation) abolish the remaining p53 activity and increase resistance to melphalan [110]. Deletions of chromosome 17p13 in *TP53* result in shorter median event-free survival (EFS) (14.6 months) and median OS (22.4 months) [114]. Increasingly accurate diagnostics of tumors in terms of damage to the *TP53* gene will facilitate therapeutic decisions that are beneficial for the patient [110].

Genetic and epigenetic changes in MM correlate with the stage of the disease. H3K9 acetylation at *c-myc* and cyclin D gene (*CCND1*) promoters increases in individual MM patients after melphalan treatment [115]. Moreover, platelet-derived growth factor BB (PDGF-BB) affects the expression of the *c-myc* gene through the *c-myc* promoter. PDGF-BB upregulates the expression of *myc* and at the same time reduces the sensitivity of cancer cells to the effects of melphalan [116]. Nevertheless, in the presence of cytostatics, further growth of neoplastic cells is observed. This is mainly due to the development of multidrug resistance. Overexpression of ATP binding cassette (ABC) transporters in the plasma membrane of MM cells contributes to the increase of MDR. A study indicated that melphalan is a glycoprotein P (P-gp) substrate [117]. Multidrug resistance protein 1 (MDR1) and baculoviral inhibitor of apoptosis repeat-containing 5 (survivin) are overexpressed, and Bcl-2-like protein 11 (Bim) is suppressed in RPMI8226 melphalan resistant cells [107]. One study compared the expression of microRNAs (miRNAs) between MM resistant and sensitive cell lines. Decreased MM cell growth induced by inhibition of miR-221/222 plus melphalan is associated with upregulation of the pro-apoptotic BBC3/ Bcl-2-binding component 3 (PUMA) protein, a miR-221/222 target, as well as with modulation of the drug influx–efflux L-type amino acid transporter 1 (LAT1 or SLC7A5) and the ABC transporter ABCC1/ multidrug resistance-associated protein 1 (MRP1) [118]. Overexpression of the long non-coding RNA linc00515 is detected in LP1 melphalan-resistant cells, indicating that linc00515 not only promotes carcinogenesis but also enhances the drug resistance of MM cells. The authors confirmed that knockdown of linc00515 inhibits autophagy and chemoresistance by upregulating miR-140-5p and downregulating autophagy related 14 (ATG14) in MM cells [119].

Interactions between MM cells and the bone marrow microenvironment may also be a source of resistance to melphalan. Increased concentrations of interleukin-6 (IL-6) induced by high-dose melphalan facilitate the survival of melphalan-resistant cells. Patients treated with high-dose melphalan, stem cell transplantation, and anti-IL-6 antibody have a better chance of survival [120]. Several inhibitors of the IL-6/Janus kinase (JAK)/ Signal transducer and activator of transcription 3 (STAT3) pathway have been investigated to reduce the proliferation of MM cells [121]. Cell-adhesion mediated drug resistance (CAM-DR) to melphalan is induced in MM cell lines and in patient primary cells through adhesion to fibronectin or bone marrow stromal cells (BMSCs), which is mediated by very late antigen-4 (VLA4) integrin (α4β1) and VLA-5 (α5β1) [122]. Suppression of integrin β7 decreases adhesion to fibronectin and E-cadherin and inhibits CAM-DR to bortezomib or melphalan in MM cells [123]. Epithelial–mesenchymal transition (EMT)-like features mediated by integrin-α8 may also contribute to melphalan resistance. The mRNA expression of the growth factor receptors platelet-derived growth factor receptor alpha (PDGFRA) and platelet-derived growth factor receptor beta (PDGFRB) is upregulated following integrin-α8 overexpression [124]. Overexpression of ATP-dependent DNA helicase Q1 (RECQ1) helicase is also a factor that protects MM cells from melphalan cytotoxicity, as shown in a group of patients with poor outcomes. RECQ helicases are involved in the maintenance of chromosome stability during replication and recombination. RECQ1 overexpression protects MM cells against bortezomib or melphalan. The comet assay showed that despite overexpression of RECQ1, melphalan induced DNA damage, although the rate of DNA repair increased over time [125].

Research suggests that oxidative stress plays a role in inducing mutations and enhancing the growth of cancer cells. Deregulation of genes involved in the response to oxidative stress is associated with poor outcomes and melphalan resistance in MM. Melphalan induces reactive oxygen species and decreases glutathione (GSH) concentration. Pretreatment with a physiological concentration of GSH protects MM cells from melphalan-induced cell cycle arrest and cytotoxicity [126].

## 8. Attempts to Find a “Better Melphalan”

The currently available melphalan therapy is associated with decreased selectivity, high toxicity, and the potential for the development of drug resistance. Side effects and the development of resistance are, in fact, the main obstacles to most existing cancer therapies. Because of numerous undesirable actions related to melphalan therapies, the introduction of new treatment regimens is essential. According to the literature, the most promising research has led to the solutions listed in the next paragraphs.

### 8.1. Drug Carriers as a Way to Reduce Systemic Toxicity

Polymer-drug conjugates play an important role in improving the targeting of cancer cells and increasing the selectivity of anti-cancer drugs. Safe and efficient drug carriers capable of delivering anti-cancer drugs specifically to their destination without causing side effects are currently sought. Loss molecular weight anti-cancer drugs are conjugated to polymeric carriers to produce a polymer-drug conjugate, which generally improves the distribution of the anti-cancer drug molecule. The main roles of polymer-drug conjugates are as follows: (1) to increase the bioavailability of the chemotherapeutic agent by increasing the water solubility of poorly soluble or insoluble drugs; (2) to protect the drugs against deactivation, and to preserve their activity during circulation; (3) to reduce the body’s immune response by decreasing the antigenic activity of the drug; and (4) to actively target the drug specifically to its site of action. In a study by Xu et al. [48], quantum dots (QDs) and melphalan were attached to a hyaluronic acid (HA) skeleton to synthesize a polymer-drug conjugate. The rate of drug release was significantly higher under acidic conditions (pH = 5.8), which simulate the microenvironment of cancer cells or tissues, than under basic conditions (pH = 7.4) [127]. HA binds specifically to various cancer cells that overexpress the CD44 receptor [128]. The advantage of HA is its property of natural degradation in the body. This process is mainly regulated by the enzyme hyaluronidase, which cleaves N-acetyl-d-glucosaminidic bonds in the HA backbone. Normal tissue is weakly alkaline (pH > 7.00), and tumor tissues and their surroundings are acidic (pH 4.5–6.0) with high expression of CD44 receptors that can direct HA-QDs-MEL towards tumor sites. Hence, the HA-QDs-MEL conjugate was stable in blood and normal tissues, and the drug was released in cancerous tissues. The HA-QDs-MEL conjugate shows excellent drug release properties, and may be a potential candidate for cancer chemotherapy with very high selectivity and low adverse effects on normal tissues [127].

To improve water solubility, systemic circulation time, and pharmacokinetic profiles, a research team led by Lu [129] synthesized and investigated a number of MEL-OCM-chitosan conjugates combined with various amino acid spacers (including glycine, l-phenylalanine, l-leucine, and 1-proline). OCM-chitosan shows no toxicity, high water solubility, biodegradability, and biocompatibility, and is thus one of the most useful candidate drug carriers. In addition, OCM-chitosan contains a large number of -COOH and-NH2 groups in the molecule that can be easily conjugated to drugs and proteins via a direct link or through a linker. MEL-OCM-chitosan conjugates show satisfactory water solubility compared with free melphalan. In vitro studies show that conjugates are stable in plasma, although they are rapidly degraded in an enzyme solution [129,130].

To solve the problems associated with the poor water solubility and rapid elimination of the drug, which reduce the specificity of melphalan, poly (amidoamine) (PAMAM) porphyrin conjugates with melphalan were synthesized and characterized. The dendrimeric conjugates show satisfactory water solubility compared with free melphalan. The size of the dendrimer plays a key role in controlling the drug content and the diameter of the melphalan conjugates. In vitro cellular cytotoxicity studies show that the dendrimeric conjugation strategy and the use of PAMAM dendritic arms as spacers improves the antitumor activity of the conjugates, which also show lower toxicity than free melphalan [131].

Melphalan-flufenamide (melflufen; L-melphalanyl-p-L-fluoro-phenylalanine ethyl) is an enzyme-activated melphalan prodrug that provides faster and greater intracellular melphalan accumulation in cancer cells. Melflufen is a newly constructed alkylating dipeptide that exhibits significantly higher anti-tumor activity than melphalan in vitro and in vivo. Chemically, melflufen is a dipeptide ethyl ester consisting of melphalan and para-fluoro-L-phenylalanine [132]. Melflufen, which is activated by hydrolytic cleavage of the peptide bond in a process that leads to high intracellular concentrations of melphalan, is capable of interacting with nucleic acids in cancer cells. Aminopeptidase N metalloprotease (APN; CD13) is directly involved in the activation of melflufen [133]. Melflufen targets tumor cells because it is a substrate for aminopeptidases that are overexpressed in cancer cells [134]. By using a simple peptide bond, melflufen activity is directed at cells expressing APN, thereby providing a peptidase-potentiated effect [132].

Melflufen transport to cells is rapid; it hydrolyzes in the cytoplasm almost immediately, forming a free and more hydrophilic form of melphalan [134]. Exposure of various tumor cells to melflufen in vitro results in at least a 10 to 20-fold higher intracellular concentration of melphalan than equimolar doses of melphalan [135]. Chauhan et al. [136] showed that melflufen is (1) 10 times more active against hematological cancer cells than melphalan; (2) blocks the migration of MM cells and inhibits tumor-associated angiogenesis; (3) induces DNA damage associated with γ-H2A histone family member X (γ-H2AX) and p53 induction; and (4) is associated with caspase activation and poly-ADP ribose polymerase (PARP) cleavage via melflufen-induced apoptosis. In vitro results were confirmed in a human MM xenograft model, which showed better inhibition of tumor growth and longer survival for melflufen than for melphalan. Melflufen causes rapid, strong, and irreversible DNA damage, which may explain its ability to overcome melphalan resistance in MM cells. Peripheral blood-derived mononuclear cells (PBMCs) are at least 10 times less sensitive to melphalan than cancer cells [132,136]. It shows high anti-tumor activity in cell lines and primary lymphoma cell cultures, as well as in a xenograft mouse model [135]. Numerous studies have also demonstrated the activity of melflufen in solid tumor cells [132,134,137,138]. Studies using solid tumor models show that melflufen induces at least a 10-fold higher melphalan load associated with high cytotoxicity against tumor cells [135]. When tested in primary cultures of human cells representing 20 different types of human malignancies, melflufen showed 50 to 100-fold greater potency than melphalan [134]. Melflufen can overcome melphalan resistance and induce synergistic anti-MM activity in combination with bortezomib, lenalidomide or dexamethasone. A recent multicenter, international, open-label, phase I–II study showed that melflufen is active in patients with relapsed and refractory MM (RRMM). These results demonstrate the feasibility of this scheme and support the initiation of additional clinical studies with melflufen in MM, both in combination with dexamethasone and in triplet with additional drug classes [139,140]. In a Phase I study, the established maximum tolerated dose was 40 mg melflufen plus dexamethasone. In Phase II, patients treated with combination therapy (meflufen + dexamethasone) achieved an ORR of 31%, achieved a clinical benefit ratio of 49%, duration of response was 8.4 months, PFS was 5.7 months, and OS was 20.7 months [141]. The phase II HORIZON clinical study (NCT02963493) is currently underway to assess the efficacy and safety of melflufen + dexamethasone in 157 patients with RRMM resistant to pomalidomide and/or daratumumab. The ongoing phase III OCEAN clinical trial (NCT03151811) is investigating the efficacy and safety of melflufen in combination with dexamethasone versus pomalidomide/dexamethasone in patients with RRMM. Eligible patients are refractory to both lenalidomide and last-line treatment and have not received prior pomalidomide. The primary endpoint is PFS and the secondary endpoints are OS, ORR, response time, and safety [141].The studies of melflufen and dexamethasone carried out recently by Richardson et al. showed clinically significant efficacy of these compounds and a manageable safety profile in patients with heavily pretreated RRMM, including those with triple-class-refractory and extramedullary disease [140]. Based on these results, in February 2021, the Food and Drug Administration approved PEPAXTO^®^ (melphalan flufenamide, also known as melflufen), in combination with dexamethasone, to treat adult patients with relapsed or refractory multiple myeloma, who received at least four prior lines of treatment and whose disease is resistant to at least one proteasome inhibitor, one immunomodulatory drug, and one CD38-directed monoclonal antibody.

### 8.2. Chemical Modifications of the Melphalan Molecule as a Way to Improve Antitumor Activity

**Figure 2:**
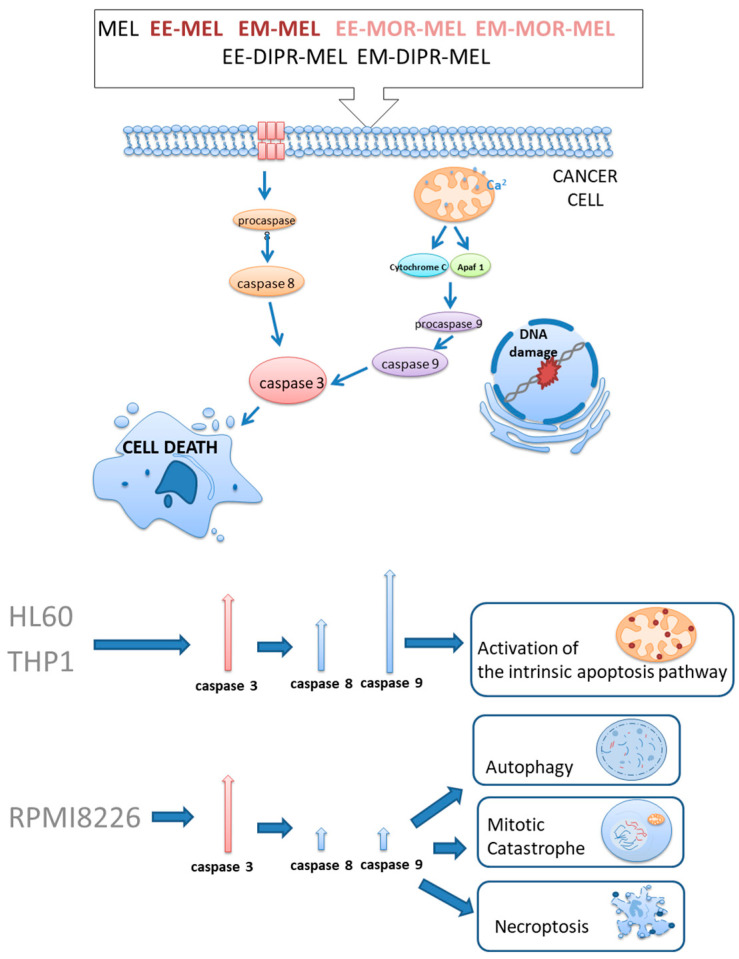
Mechanism of action of melphalan and new derivatives of melphalan. The cellular response to the test compounds varied depending on the cell type. Melphalan and its analogues activate the intrinsic pathway of apoptosis in acute monocytic leukemia cells and promyelocytic leukemia cells. In MM cells, these compounds activate mechanisms of cell death other than apoptosis, such as mitotic disaster, autophagy, or necroptosis. Figure is adapted from previous publication [142].

The structure of the melphalan molecule is noteworthy because of the presence of two modifiable functional groups: a carboxyl group and an amino group. These modifications provide extensive comparisons. Gajek et al. [142] synthesized and investigated new melphalan analogues modified in both functional groups. The resulting compounds are methyl and ethyl esters of melphalan (EE-MEL/EM-MEL), followed by melphalan esters also modified with a morpholine ring (EE-MOR-MEL/EM-MOR-MEL) or a dipropylene chain (EE-MOR-MEL/EM-DIPR-MEL). The derivatives were used to assess the potential antitumor properties of the structural changes compared with melphalan. The study was performed using three models of hematological malignancy: RPMI8226 (myeloma cancer cells), THP1 (acute monocytic leukemia cells), and HL60 (promyelocytic leukemia cells). Modification of the carboxyl group by esterification of the compound showed the highest efficacy, and the results indicated that the ester group is necessary to increase the cytotoxic activity of melphalan [142,143]. In vitro studies conducted by this group of researchers showed that new MEL analogues have better antitumor activity than the parent drug. The compounds are characterized by high cytotoxicity and genotoxicity. Determining the potential ability of melphalan derivatives to activate cysteine proteases (caspase-3, -8, and -9), a characteristic mechanism in the course of programmed cell death, is an important element of the study, because the ability of drugs to induce apoptosis is considered an important criterion for assessing their therapeutic efficacy. This is the preferred type of cell death, as it is a physiological process that does not cause inflammation. The cellular response to the test compounds varies depending on the cell type. In MM cells, these compounds activate mechanisms of cell death other than apoptosis, such as mitotic disaster, autophagy, or necroptosis. Furthermore, the most promising derivatives (EE-MEL, EM-MEL, EM-MOR-MEL) were selected to assess the cytotoxic effects of these compounds on normal cells, namely, PBMCs. Chemical modifications, in particular esterification, can increase the antitumor activity of melphalan by increasing the lipophilicity of the drug. However, this study is limited to in vitro data only and must be verified by in vivo experiments [142] (Figure 2).

## 9. Conclusions

The use of high dose melphalan was first described by McElwain and Powles in 1983 [46]. After 37 years of its application in the treatment of MM therapy, the drug remains a part of treatment regimens. High-dose melphalan and ASCT are safe in patients with MM. Despite the current difficulty in accessing oncology centers, work on the development of further MM therapies has not stopped. Here, we described the use of therapy involving combinations of drugs with different mechanisms of action, including alkylating agents, immunomodulatory drugs, histone deacetylase inhibitors, proteasome inhibitors, and monoclonal antibodies. We showed that the use of various drugs is effective in the fight against MM. Changing the structure of melphalan by modifying the carboxyl and amino groups, as well as creating transporters for melphalan, are more effective strategies for the treatment of MM than the use of the unmodified melphalan molecule. The new analogues are characterized by higher cytotoxicity and genotoxicity, or the ability to induce apoptosis in hematological malignancies, and thus represent an important step in finding an effective anti-cancer therapy.

## Data Availability

Not applicable.
